# Baseline Survey of Root-Associated Microbes of *Taxus chinensis* (Pilger) Rehd

**DOI:** 10.1371/journal.pone.0123026

**Published:** 2015-03-30

**Authors:** Qian Zhang, Hongwei Liu, Guiling Sun, Iain W. Wilson, Jianqiang Wu, Angela Hoffman, Junwen Cheng, Deyou Qiu

**Affiliations:** 1 State Key Laboratory of Tree Genetics and Breeding, The Research Institute of Forestry, Chinese Academy of Forestry, Beijing 100091, China; 2 Key Laboratory of Economic Plants and Biotechnology, Kunming Institute of Botany, Chinese Academy of Sciences, Kunming 650201, China; 3 CSIRO Agriculture Flagship, Canberra ACT 2001, Australia; 4 Department of Chemistry, University of Portland, OR 97203, United States of America; 5 Key Laboratory of Biological and Chemical Utilization of Forest Resources, Zhejiang forestry Academy, Hangzhou 310023, China; Dong-A University, KOREA, REPUBLIC OF

## Abstract

Taxol (paclitaxel) a diterpenoid is one of the most effective anticancer drugs identified. Biosynthesis of taxol was considered restricted to the *Taxus* genera until Stierle *et al*. discovered that an endophytic fungus isolated from *Taxus brevifolia* could independently synthesize taxol. Little is known about the mechanism of taxol biosynthesis in microbes, but it has been speculated that its biosynthesis may differ from plants. The microbiome from the roots of *Taxus chinensis* have been extensively investigated with culture-dependent methods to identify taxol synthesizing microbes, but not using culture independent methods.,Using bar-coded high-throughput sequencing in combination with a metagenomics approach, we surveyed the microbial diversity and gene composition of the root-associated microbiomefrom *Taxus chinensis* (Pilger) Rehd. High-throughput amplicon sequencing revealed 187 fungal OTUs which is higher than any previously reported fungal number identified with the culture-dependent method, suggesting that *T*. *chinensis* roots harbor novel and diverse fungi. Some operational taxonomic units (OTU) identified were identical to reported microbe strains possessing the ability to synthesis taxol and several genes previously associated with taxol biosynthesis were identified through metagenomics analysis.

## Introduction

Taxol (paclitaxel), a complex diterpenoid first isolated from the bark of pacific yew tree (*Taxus brevifolia*), is widely used in chemotherapy treatment of lung, ovarian and breast cancer [1, 2]. The supply of taxol is currently constrained and supplied by a number of routes including harvesting from relatively slow-growing *Taxus* trees [3]. Thus, alternative sources for taxol have been actively explored for the past 20 years, including a search for taxol-producing microorganisms [4].

Fungal endophytes are well known sources of diverse biologically active secondary metabolites, with a number of applications as pharmaceutical products. In 1993, Stierle and colleagues discovered that an endophytic fungus from *Taxus brevifolia* could independently synthesize taxol [5]. This groundbreaking work resulted in the identification of a large number of endophytes isolated from *Taxus* species [6] and other medicinal plants [7–9], and the study of their ability to synthesize taxol and other chemicals with therapeutic uses. Other than fungi, several bacterial strains were subsequently found to have the capacity to produce taxol [10, 11] (S1 Table).

Potential advantages of microbial taxol production include fast growth at high cell density, relatively easy genetic manipulation, and the possibility of scaling up to an industrial level [12]. Current research on microbe-related taxol-production focuses on screening taxol-producing endophytic microbes [5], heterologous expression of taxol precursors in microorganisms [13] improving taxol yield by genome shuffling [14], genetic engineering [15], and process optimization [16].

Many studies have focused on biosynthesis of taxol. In *Taxus*, the biosynthetic pathway of taxol has been clearly elucidated, consisting of 13 genes (S2 Table). There have been several reports focusing on the molecular basis of taxol-production in microorganisms; however, little is known about the synthesis mechanism of taxol in microbes. *Taxus*-derived genes or their fragments responsible for taxol synthesis have been used as molecular probes for the screening of microorganisms [17]. Several genes that encode the corresponding taxol pathway enzymes previously found in *Taxus* spp. were reported to exist in endophytic fungi [18–21]. However, studies also showed that existence of these genes does not guarantee the ability to synthesize taxol. For example, among 12 endophytic fungal strains containing the taxadiene synthase gene (*TS*), which encodes a rate-limiting enzyme in the taxol biosynthetic pathway in *Taxus*, only 3 strains could synthesize taxol [22]. Even in the strains that possess a functional *TS* gene, the ability to synthesize the precursor for taxol has not been verified. It has been speculated that the biosynthesis pathway of taxol in microbes is different from that in *Taxus* [17], which is supported by the finding that candidate taxol biosynthetic genes from the taxol synthesizing *Penicillium aurantiogriseum* NRRL 62431 were significantly different and had evolved independently from plants [23].

Next generation sequencing technologies have enabled metagenomic and metagenetic analysis of soil microorganism species and gene composition of microbiota [24, 25]. However, there are currently no studies characterizing species and gene composition of root associated microbiome of the roots from *Taxus*. In this study, we used bar-coded high-throughput sequencing with primers targeting the 16S and 18S rRNA genes to survey root associated bacterial and fungal diversity of *Taxus* root, in conjunction with a metagenome approach to survey microbial species and gene composition in its root associated microbiome. We also studied genes putatively associated with taxol biosynthesis in the *Taxus* root associated microbiome to estimate the prevalence of taxol biosynthetic genes in the root associated microbiome.

## Results and Discussion

The aim of our study was to investigate the root-associated microbiome from *Taxus* using next generation sequencing to sequence 16S and 18S amplicons derived from *Taxus* roots. To enrich for microbial endophytes, roots were sampled fresh and their surfaces rigorously washed to remove external microflora. The isolated DNA was subjected to amplification using oligonucleotides that were designed to specifically amplify 16S (V5F-V3R) and 18S (EF4–518). Microbes were tentatively identified to OTUs using sequence homology to known species present in the NCBI database. These results must be treated with caution, as most matches were not to type strains, and therefore there is the possibility of incorrect identification.

**Fig 1:**
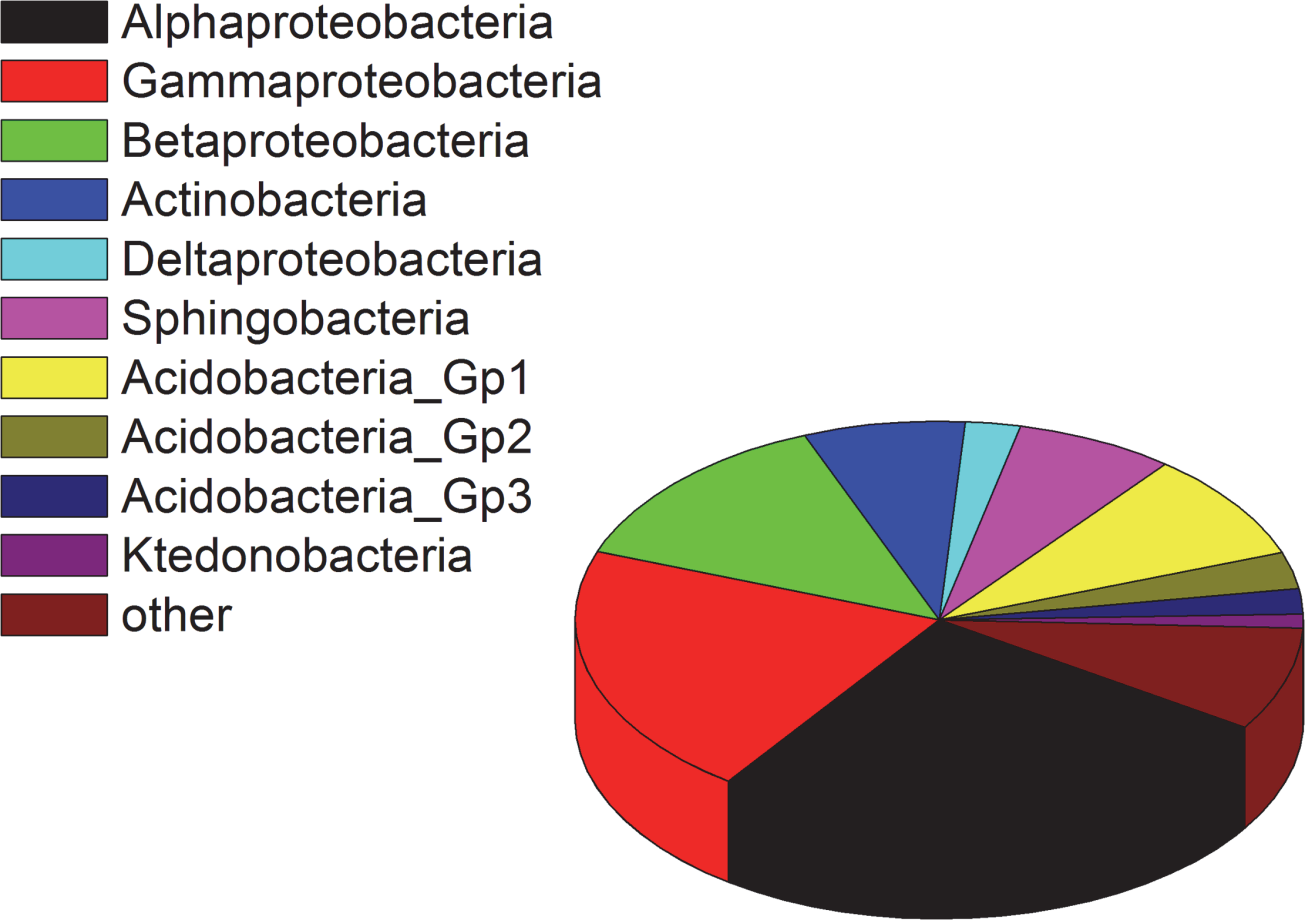
Bacterial diversity as a percentage associated with root endophyte of *Taxus chinensis* (Pilger) Rehd.—using 16s pyro-sequencing.

From the 16S bacterial library, a total of 24,750 sequences were obtained with the V5F-V3R primer set. Only high quality sequences consisting of 20,538 sequences with a length distribution around 480–530 bp were used for analysis. In total, 913 OTUs were identified based on 97% sequence similarity. The majority of these OTUs were from Proteobacteria (63.24%), Acidobacteria (14.35%), Bacteroidetes (7.83%) and Actinobacteria (7.18%). Of the 21 most abundant OTUs, 15 were from Proteobacteria (Table 1). At the class level, the OTUs were mainly from Alphaproteobacteria (25.67%), Gammaproteobacteria (20.75%) and Betaproteobacteria (13.38%) (Fig 1). These 913 OTUs consisted of 158 genera. Shannon index of 16S sequences data was 7.25, Chao1 was 1918.57, and the PD_whole_tree was 51.55. In the U.S. Pat. No. 5,561,055, there is one bacterium disclosed, which was referred to as *Erwinia taxi*, for the production of taxol (later characterized as *Sphingomonas taxi*), which was isolated from *Taxus canadensis*. From our data, three OTUs (accounting for 0.24% of the total sequences) from the genus *Sphingomonas* were identified in the root of *Taxus chinensis*. BLASTn analysis to GenBank indicated that none of these three OTUs matched any reported species that have taxol-producing capacity.

**Table 1:** GenBank accession number, taxonomy, and reads number of the 21 most abundant OTUs in 16S pyrosequencing.

ID	GenBank #	% Identity	Match	Phylum	Class	Order	Family	Reads No.
TR871	JF833490	99	Uncultured Steroidobacter sp.	Proteobacteria	Gammaproteobacteria	Xanthomonadales	Sinobacteraceae	807
TR568	JF958143	99	Burkholderia sp.	Proteobacteria	Betaproteobacteria	Burkholderiales	Burkholderiaceae	764
TR621	JX424780	100	Bradyrhizobium elkanii	Proteobacteria	Alphaproteobacteria	Rhizobiales	Bradyrhizobiaceae	590
TR764	EF075691	99	Uncultured gamma proteobacterium clone	Proteobacteria	Gammaproteobacteria			342
TR494	AB808756	100	Streptomyces sp.	Actinobacteria	Actinobacteridae	Actinomycetales	Streptomycineae	327
TR679	CP005950	100	Rhizobium etli	Proteobacteria	Alphaproteobacteria	Rhizobiales	Rhizobiaceae	211
TR430	EF665802	98	Uncultured gamma proteobacterium clone	Proteobacteria	Gammaproteobacteria			204
TR087	EF075624	100	Uncultured Acidobacteria bacterium clone	Acidobacteria				202
TR035	EF075887	98	uncultured alpha proteobacterium	Proteobacteria	Alphaproteobacteria			180
TR026	FJ570455	98	Uncultured gamma proteobacterium clone	Proteobacteria	Gammaproteobacteria			165
TR509	EF072247	99	uncultured Acidobacteriaceae bacterium	Acidobacteria	Acidobacteriales	Acidobacteriaceae		162
TR658	KF150437	100	Dyella marensis	Proteobacteria	Gammaproteobacteria	Xanthomonadales	Xanthomonadaceae	153
TR045	JX545161	99	Uncultured Caulobacter sp.	Proteobacteria	Alphaproteobacteria	Caulobacterales	Caulobacteraceae	143
TR405	EF075573	99	Uncultured Sphingobacteriales bacterium clone	Bacteroidetes	Sphingobacteriia	Sphingobacteriales		126
TR383	GU047629	99	uncultured Caulobacteraceae bacterium	Proteobacteria	Alphaproteobacteria	Caulobacterales	Caulobacteraceae	124
TR123	EU440697	99	uncultured Rhodospirillaceae bacterium	Proteobacteria	Alphaproteobacteria	Rhodospirillales	Rhodospirillaceae	110
TR647	AY673350	99	Acidobacteria bacterium Ellin7184	Acidobacteria				105
TR897	EF075729	99	uncultured Rubrivivax sp.	Proteobacteria	Betaproteobacteria	Burkholderiales	Rubrivivax	105
TR231	HQ882705	99	Duganella sp.	Proteobacteria	Betaproteobacteria	Burkholderiales	Oxalobacteraceae	104
TR315	JQ701563	97	uncultured Phyllobacteriaceae bacterium	Proteobacteria	Alphaproteobacteria	Rhizobiales	Phyllobacteriaceae	96
TR118	EF072359	97	uncultured Flavobacteriia bacterium	Bacteroidetes	Flavobacteriia			92

**Fig 2:**
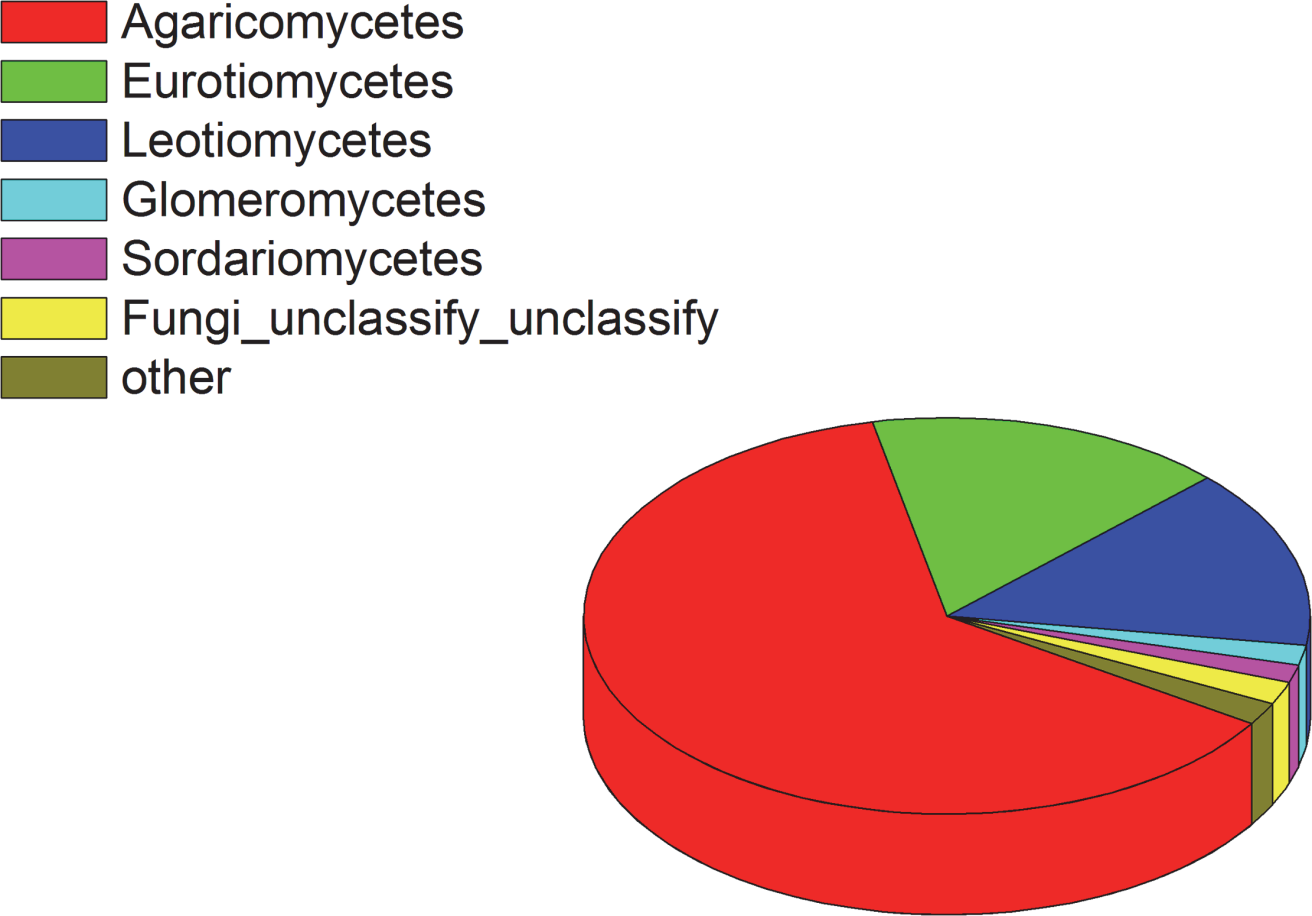
Fungal diversity as a percentage associated with root endophyte of *Taxus chinensis* (Pilger) Rehd.—using 18s pyro-sequencing.

Through analysis of the sequences obtained from the 18S-derived library, we identified a total of 110,272 sequences obtained with the primer set EF4–518. A total of 34,739 reads were included for analysis after filtering for quality. The average length of these high quality reads was 363 bp. In total, 187 OTUs were defined based on the 97% sequence similarity criteria. These OTUs mainly belonged to Basidiomycota (62.624%) and Ascomycota (33.018%). From the 20 most abundant OTUs, 12 were from Basidiomycota (Table 2). At the Class level, the majority of OTUs were from Agaricomycetes (62.55%), Eurotiomycetes (16.00%) and Leotiomycetes (14.66%) (Fig 2). These 187 OTUs consisted of 69 genera. Shannon index of 18S sequences data was 3.90, chao1 was 252.62, and the PD_whole_tree was 8.41. Five genera were found to contain reported species with taxol production capacity, *Aspergillus* (1 OTU, 22 sequences, 0.063% of total sequences), *Bionectria* (1 OTU, 3 sequences, 0.009% of total sequences), *Cladosporium* (1 OTU, 39 sequences, 0.112% of total sequences), *Alternaria* (1 OTU, 100 sequences, 0.288% of total sequences) and *Pestalotiopsis* (1 OTU, 1 sequence, 0.003% of total sequences). Sequence similarity analysis using BLASTn against the GenBank nucleotide sequences showed that sequence of the OTU from *Alternaria* genus was highly similar to the fungal strain *Alternaria* sp. Tax-4 that possesses taxol-production capacity (Accession No. KF193057, with a 99% query cover, 99% identity, e-value = 0.0).

**Table 2:** GenBank accession number, taxonomy, and reads number of the 20 most abundant OTUs in 18S pyrosequencing.

ID	GenBank #	% Identity	Match	Phylum	Class	Order	Family	Reads No.
63	DQ873608	99	Hyphodontia barba-jovis isolate 2037a	Basidiomycota	Agaricomycetes	Corticiales	Corticiaceae	8188
150	DQ440644	99	Hemimycena gracilis	Basidiomycota	Agaricomycetes	Agaricales	Tricholomataceae	4374
1	EF024609	95	Uncultured Boletaceae clone	Basidiomycota	Agaricomycetes	Boletales		4284
46	FJ358327	99	Chaetothyriales sp. TRN247	Ascomycota	Eurotiomycetes	Chaetothyriales		3957
142	JN938729	99	Phialocephala sp.	Ascomycota	Leotiomycetes	Helotiales	mitosporic Helotiales	3196
6	DQ898724	99	Sistotrema athelioides	Basidiomycota	Agaricomycetes	Corticiales	Corticiaceae	1987
145	DQ520103	99	Craterocolla cerasi	Basidiomycota	Agaricomycetes	Sebacinales	Sebacinaceae	1405
34	GQ330619	99	Uncultured Helotiales clone	Ascomycota	Leotiomycetes	Helotiales		1175
70	DQ444856	99	Hydropus marginellus	Basidiomycota	Agaricomycetes	Agaricales	Tricholomataceae	618
12	JQ926736	99	Hohenbuehelia sp.	Basidiomycota	Agaricomycetes	Agaricales	Pleurotaceae	524
181	GQ404741	99	uncultured Ascomycota	Ascomycota				492
175	HQ661373	99	Uncultured Scytalidium	Ascomycota				475
148	JN940455	99	Macrolepiota mastoidea	Basidiomycota	Agaricomycetes	Agaricales	Agaricaceae	350
70	DQ444856	99	Hydropus marginellus	Basidiomycota	Agaricomycetes	Agaricales	Tricholomataceae	331
68	JN939906	100	Coprinellus congregatus	Basidiomycota	Agaricomycetes	Agaricales	Psathyrellaceae	299
169	JN940003	97	Polyporus cf. grammocephalus 1 KH-2011	Basidiomycota	Agaricomycetes	Polyporales	Polyporaceae	167
36	HQ840409	99	Ilyonectria radicicola	Ascomycota	Sordariomycetes	Hypocreales	Nectriaceae	164
162	JX158869	94	uncultured Eupenicillium	Ascomycota	Eurotiomycetes	Eurotiales	Trichocomaceae	155
67	DQ440644	99	Hemimycena gracilis	Basidiomycota	Agaricomycetes	Agaricales	Trichocomaceae	135
176	FJ358327	100	Chaetothyriales sp.	Ascomycota	Eurotiomycetes	Chaetothyriales		135

The microbiome colonizing the root surface and the endophytic compartment (within the root) contribute to plant growth, productivity, carbon sequestration and secondary metabolite biosynthesis [6–8]. The high throughput next generation sequencing technologies together with the bioinformatic pipelines have enabled the description of culture-independent microflora associated with numerous environmental and human microbiomes and to reveal meta-genomic composition. For example, sequencing of the bacterial 16S ribosomal RNA gene showed different bacterial communities are strongly influenced by soil type, and some bacteria vary quantitatively between plants of different developmental stage and genotype [24]. The root-associated microbiome from fresh roots of *T*. *chinensis* appear to be associated with a broad spectrum of endophytic microbial taxa, and phylotypes representing a number of phyla. Some taxa that were ubiquitous across *Taxus* plants, such as *Aspergillus* and *Hypocrea*, have also been observed in previous studies focusing on microbial diversity of *Taxus* [6]. Our study revealed a higher species richness and diversity than previous studies (e.g., 29 fungal isolates in Xiong et al. 2013 [17]). Diversity of endophytic fungi of *Taxus* spp. have been explored by isolating culturable fungi on PDA (Potato dextrose agar) and SMA (Sabouraud Maltose Agar) culture medium [6,17]. A number of fungi have been reported as endophytes in different *Taxus* species. Caruso et al. (2000) isolated and identified 25 different genera in *T*. *baccata* [26]. Wang et al. (2008) found 5 genera and 3 unidentified fungi in *T*. *mairei* [27]. Liu et al. (2009) reported 26 genera in *T*. *chinensis* [28]. Rivera-Orduña et al. (2011) found 29 fungal isolates in 24 genera in *T*. *globosa* (Mexican yew) [6]. Xiong et al. (2013) found 29 fungal isolates in 8 genera in *T*. *media* [17]. However, non-culturable endophytic isolates cannot be found using these conventional methods. To overcome this problem, in this study we used high-throughput sequencing technology on libraries derived from 16S and 18S amplicons from freshly isolated DNA samples, and found it more powerful and more efficient than traditional morphological identification and Sanger sequencing in characterizing community structure. Our high-throughput amplicon sequencing revealed 187 fungal OTUs which is higher than any previously reported fungal number identified with the culture-dependent method [6,17,26,27,28], suggesting that *T*. *chinensis* roots harbor novel and diverse fungi.

It should be noted that most fungi reported as endophytes in *Taxus* have been identified as ascomycetes and their anamorphs. Basidiomycetous endophytes have only been reported in limited number of studies. For example, the fungal isolates belonged to Ascomycota (77.2%) and Basidiomycota (22.8%) in Rivera-Orduña et al. (2011) study [6]. All the fungal isolates belonged to Ascomycota (100%) in Xiong et al. (2013) [17]. Our results show that the majority of the fungal OTUs belonged to Basidiomycota (62.624%). Previous studies have also showed *Penicillium* and *Hopoxylon* [6], *Colletotrichum* and *Glomerella* [17] are dominant genera. However, these 4 genera were not detected in our study. We found *Hyphodontia* (24.713%), *Hemimycena* (12.994%), *Phialocephala* (9.243%) are the three dominant genera, and to our knowledge, this is the first time these three genera were reported in any *Taxus* sp. The difference between our results and Rivera-Orduña et al (2011) [6] or Xiong et al (2013) [17] may be due to tissue specificity, as we used fresh root as biological samples in this study, while bark, branches, leaves and roots were used in Rivera-Orduña et al (2011) [6] and bark pieces and leaves were used in Xiong et al. (2013) [17].

Plants are hosts of a variety of microbes including fungi and bacteria. Bacteria possess a higher rate of metabolism than fungi. It was expected that larger quantities of taxol could be extracted in shorter periods from bacteria. However, only one bacterium, *Erwinia taxi* has been reported to possess the capacity to synthesize taxol [29]. It would be highly desirable to find other bacteria having highly metabolic capacities isolated from different species of *Taxus* for the production of taxol and related taxanes. In addition, some strains (e.g. *Moraxella* sp., *Bacillus macerans*, *Bacillus circulans*, and *Micrococcus* sp.) had been reported to be able to remove the xylosyl group from 7-xylosyltaxanes, an important step in taxol semi-synthesis [30].

Using clone libraries, Gammaproteobacteria, Betaproteobacteria, and Actinobacteria were found to be more abundant in the rhizosphere of *T*. *media* from the temperate region, and Acidobacteria was more abundant in the subtropical *Taxus mairei* rhizosphere [31]. In our study, Actinobacteria and Acidobacteria were also abundant phyla, with Proteobacteria being the most abundant phylum. Proteobacteria is widespread in natural ecosystems of plant species. For various pine forest soils in British Columbia, Proteobacteria contributed to about 50% of the total clone library [32]. Filion et al. (2004) determined that the majority of 16S rRNA gene clones obtained from the rhizosphere of healthy spruce seedlings grouped with the Proteobacteria (27%) [33]. Our study is the first report of Proteobacteria being dominant in the root of *T*. *chinensis*.

**Fig 3:**
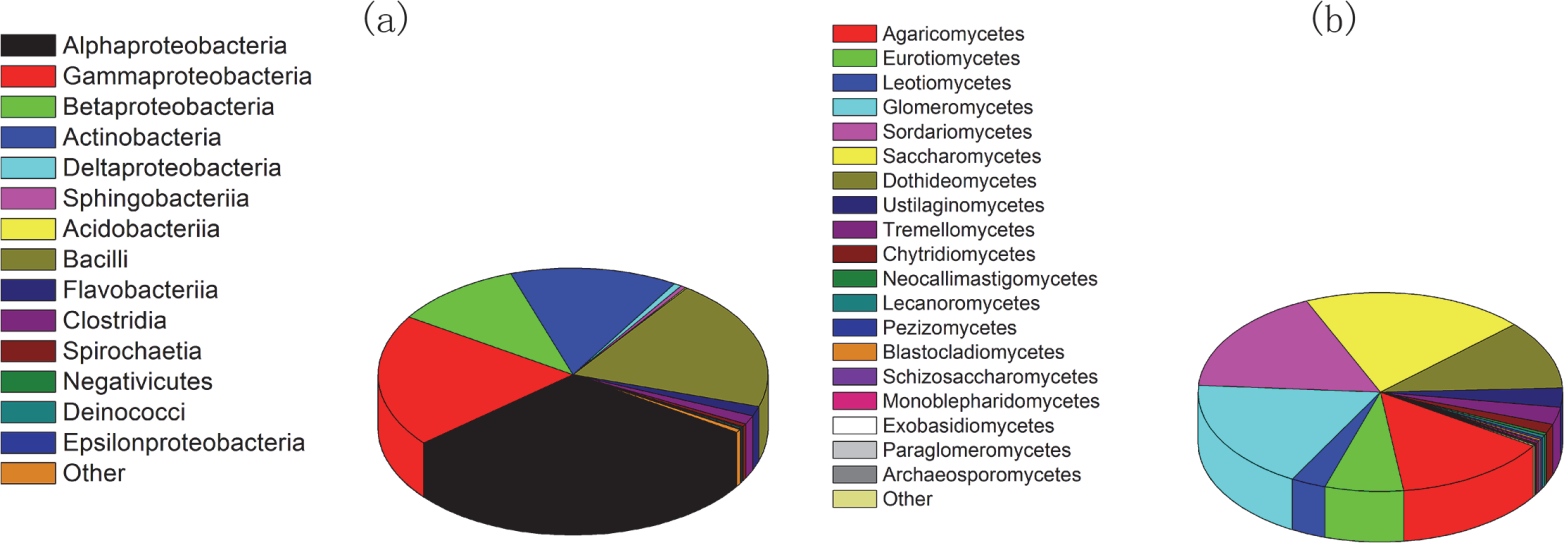
Bacterial and fungal diversity as a percentage associated with root endophyte of *Taxus chinensis* (Pilger) Rehd—using meta-genome sequencing. a: bacterial; b: fungal.

Metagenomic analysis that consisted of sequencing a DNA library derived from the *T*. *chinensis* root DNA showed that Alphaproteobacteria, Gammaproteobacteria and Bacilli were the most dominant bacterial phyla, and Saccharomycetes, Glomeromycetes and Sordariomycetes were the dominant fungi (Fig 3). Five bacterial genera (Erwinia, Curtobacterium, Pantoea, Bacillus and Sphingomonas) were reported to have species with taxol-production capacity, accounting for 14.9% of total contigs (S3 Table). Thirty-six fungal genera were found to have reported species with taxol-production capacity (S4 Table). Five species with known taxol-production capacity (*Colletotrichum gloeosporioides*, *Guignardia mangiferae*, *Fusarium solani*, *Aspergillus flavus*, *Pestalotiopsis microspora*) were identified by our metagenomic sequencing work (Fig 3).

In our metagenomic analysis, we obtained 20,267.65 MB bp (around 20 G) data. Sequences from host plant were filtered using SOAPaligner (Version 2.21, http://soap.genomics.org.cn/soapaligner.html) with a match requirement of 95% sequence identity to the transcriptome sequences of *Taxus chinensis* and we got 323.5 Mbp clean data. Therefore, the percentage of microbes DNA in our metagenomic library is 1.6% and the percentage of DNA contamination of plant genes is 98.4%. IDBA_UD assembly identified 386,581 genes using metagenomic analysis. There were 634 genes (S5 Table) similar to those known to participate in taxol synthesis in *Taxus*, with protein sequence identities ranging from 24.11% to 98.8% (S5 Table). Similar sequence identities have been reported from other taxol-producing fungi [17]. Notably, one gene (gene_TR2_205230) was found to be similar to the gene for taxadiene synthase (TS) in *Taxus mairei* (accession No. ABW82998.1), with max score = 112, total score = 112, query cover = 81%, E-value = 4e-27, identity = 47.37%), which might be a key and rate limiting gene for the biosynthesis of taxol in *Taxus* root endophytic microbes.

Metagenomic analysis of the 16S and 18S sequences aided in identification of different microbial compositions (Figs 1, 2, and 3). 16S rDNA sequencing detected 10 unambiguous classes of endophytic microbes, and identified Alphaproteobacteria, Gammaproteobacteria and Betaproteobacteria as the 3 most abundant classes (Fig 1). 18S rDNA pyrosequencing detected 6 unambiguous classes of endophytic microbes, and showed Agaricomycetes, Eurotiomycetes and Leotiomycetes to be the three most abundant classes (Fig 2). However, metagenomic analysis detected 14 bacterial classes and 19 fungal classes respectively representing different dominant classes (Fig 3). This may be partially due to a deeper sequencing and our metagenomic analysis, as we obtained 20G data which is larger than 16s and 18S rDNA pyrosequencing. This would be expected, since the library preparation for the two analyzes are very different (16S and 18S analyses enriching microbial sequences due to primer specificities). These 16S and 18S metagenomics methodologies may enrich for different sequences (species), suggesting that different methodologies should be used to achieve comprehensive microbiome surveys.

In the yew tree, taxol biosynthesis involves 19 enzymatic steps from the universal diterpenoid precursor geranylgeranyl diphosphate (GGPP) produced by the plastidial methyl erythritol phosphate pathway [34]. Several reports have suggested that endophytic fungi contain genes encoding the pathway enzymes previously identified in *Taxus* spp. [18–21, 35, 36]. The reported presence of previously identified taxol genes of *Taxus* spp. in endophytic fungi were based on the results of PCR experiments using primers designed according to the published sequences of taxol biosynthetic genes from *Taxus* trees. The sequences they provided indicated that the fungal amplicons were virtually identical to the *Taxus* clones [19,20] and this lead to speculation that horizontal gene transfer occurred between microbes and *Taxus* plant [19,20]. However, recently it was shown that taxol biosynthesis was possible for *P*. *aurantiogriseum* NRRL 62431 and that putative taxol biosynthetic genes identified by whole genome sequencing were quite different from those in hosts *C*. *avellana* and *T*. *baccata* in terms of amino acid sequences, and may evolved independently [23]. Our metagenomic analysis showed one gene shared 47.37% identity with cDNA of TS from *Taxus mairei* (accession no. ABE82998.1) (Max score = 112, query cover = 81%, E-value = 4e-27). This result is consistent with Xiong et al. (2013) that the *TS* gene from the fungi shares low similarity with that from *Taxus* plant [17] and the genome sequencing of *P*. *aurantiogriseum* NRRL 62431 [23]. Therefore, it is likely that many of the highly similar taxol biosynthetic genes identified in the past few years from microbes are due to contamination from plant DNA during endophyte preparations.

Studies have shown that microbes can interact with each other or with host plants to affect taxol production [37, 38]. Addition of endophytic fungi (*Fusarium mairei*) culture broth (EFCB) in cell suspension cultures of *Taxus cuspidata* resulted in a greater than 2 fold yield than that in cultures of plant cell or endophytic fungi alone [38]. Taxol-producing endophytes may change the transcription of plant taxol biosynthetic genes and thus influence taxol content of intact *Taxus* plants and/or tissues [39] Resident fungi within a host plant could interact with one another to stimulate taxol biosynthesis, either directly or through their metabolites. Co-culture of SSM001 (an endophyte that was proposed to produce taxol), with a bark fungus (*Alternaria* or *Phomopsis*) caused a 3 to 8 fold increase in taxol production [40].Our survey of the endophytic community composition on *T*. *chinesensis* provides a starting point for analyzing interactions between endophytes, and also the interaction between endophytes and their host plant. Considering that many endophytes are pathogens or symbiont of host plants, our baseline survey of root endophytic microbes of *Taxus* plant can be helpful for disease control, cultivation management and taxol production by *Taxus* plants. Given the importance of the root-associated microbiome and the current lack of information about these communities, metagenetic analyses such as the one we described here may be warranted for other agronomically important plant species.

### Conclusions

Taxol is currently commercially obtained from a number of routes including; plantation yew trees, semisynthetic synthesis from an intermediates such as baccatin III or 10-deacetyllbaccatin III found in renewable needles of *Taxus*, or plant cell cultures [41]. Despite early optimism, taxane synthesis from endophytic taxol-producing microbes has not been economical due to low and variable yields. Our study begins a large-scale identification of candidate genes involved taxol biosynthesis in the root endophytes of *Taxus* using a metagenomics approach. Surveying varied microbiome from *Taxus* spp with culture independent techniques may provide a way to improve the metabolic engineering of taxol biosynthesis in culturable microbes by identifying superior taxol biosynthesis genes that could be inserted into non-taxol synthesizing hosts. Alternatively it may be possible to inoculate *Taxus* species with unculturable taxol synthesizing microbes to enhance taxol yields from these trees, or select for more culture amenable variants from normally unculturable taxol synthesizing species.

Our study has revealed a rich diversity of mictobes in the *Taxus* root endophytic microbiome with some OTUs identified identical to reported microbe strains possessing taxol-synthesis abilities. Metagenomics analysis confirmed that the taxol biosynthetic pathway may differ between these microbes and *Taxus*, indicating that taxol biosynthesis in *Taxus* root endophytes may have evolved independently. Our findings can shed new light on biodiversity of endophytes in *Taxus* root and how taxol-producing endophytes synthesize taxol, and will facilitate metabolic engineering for the industrial production of taxol from microbes.

## Methods

### Root sampling and DNA extraction

Plant roots were carried out on private land with the permission granted by Mr. Mingyun Yin. One 5-year—old live plants of *Taxus chinensis* (Pilger) Rehd. was collected in June 2013 from Fengxin County (114°45`E, 28°34`N) in Jiangxi province of China. The taxol-producing capability of this plant was comfired by LC-MS method. The annual average temperature is 17.3°C, and annual rainfall is 1612 mm in the site. To study the root-associated microbiome, fresh roots of *Taxus chinensis* (Pilger) Rehd. were harvested, sealed in plastic bags placed in a car refrigerator, immediately transported to the laboratory, washed with tap water, and then rinsed three times with sterile distilled water to remove external root microbiome. To remove fungal spores or hyphae (e.g. arbuscular mycorrhizal funi) on the root surface, roots were sonicated at low frequency for 3 min (30-s bursts followed by 30-s rests performed three times). Genomic DNA was then extracted from fine fresh roots (0.2 g) with Fungal DNAout Kit (TIANDZ, Beijing, China). The extracted DNA was dissolved in 50 μL TE buffer, quantified by spectrophotometry and stored at -20°C for further use.

### 16S and 18S rRNA gene amplicon preparation

Primer set V5F (forward: TCACGTACTA+CCGTCAATTCMTTTGAGTTT—V3R (reverse: ACTCCTACGGGAGGCAGCAG) and EF4 (forward: GGAAGGGG/AT GTATTTATTAG)− 518 (reverse: ATTACCGCGGCTGCTGG) were used to amplify 16S and 18S rRNA gene fragments respectively. Primer set V5F-V3R amplified the variable V3-V5 region of all bacteria. Primer set EF4-518 targeted 18S rDNA of all fungi. To perform 454 pyrosequencing, DNA primer sequences first adapted with 454 Life Science A or B sequencing adapters (19 bp), then fused with 8-bp barcoded primers: Primer B (GCCTTGCCAGCCCGCTCAG) + barcode + forward primer and Primer A (GCCTCCCTCGCGCCATCAG) + reversed primer. PCR was performed in 50-μL reaction mixtures containing 1.25 mM deoxynucleoside triphosphate, 2μL (15μM) primers, 2U Taq DNA polymerase (TaKaRa, Japan), and 50 ng (1:l) genomic DNA as template. PCR procedures were as follows: 35 cycles (95°C for 45 s, 58°C for 45 s, and 72°C for 1 min) with a final extension at 72°C for 7 min. PCR reaction mixtures were purified using QIAquick PCR Purification kit (QIAGEN), and quantified using a NanoDrop ND-1000 photometer (Thermo Scientific, USA).

### Metagenome library preparation

DNA library preparation followed the manufacturer’s instruction (paired-end sample preparation guide, Illumina). The base-calling pipeline (version Illumina Pipeline-0.3) was used to process the raw fluorescent images and call sequences.

### Pyrosequencing platform

16S and 18s amplicons were sequenced on the Roche 454 GS Titanium FLX platform and WGS DNA was sequenced on Illumina platform according to the manufacturer’s specifications.

### Processing of 16S pyrosequencing data

Sequences were trimmed for those below quality score of 25 and 200 bp in length using Quantitative Insights Into Microbial Ecology (QIIME) pipeline (http://qiime.org/scripts/split_libraries.html). The remaining sequences were assigned to samples based on unique 8-bp barcodes. Sequences were binned into Operational Taxonomic Units (OTUs) with 97% identity threshold. Each representative sequence was assigned a taxonomy using the RDP classifier [42] trained on the 4 February 2011 Greengenes reference sequences. Once OTUs were assigned taxonomy, all OTUs annotated as chloroplasts, *Viridiplantae* or *Archaea* were removed from the OTU table, resulting in the set of usable OTUs. Representative sequences of the most 20 abundant OTUs were then searched for the best BLAST hit on NCBI. A series of subsets of each library in different sizes (10, 110, 210, 310 with a step of 100) with 10 replicates were used to calculate diversity and richness indices. Representative sequence that was assigned to previously reported genus with taxol-production capacity species (S1 Table) was blasted in GenBank for further species level taxonomy identification.

### Processing of 18S pyrosequencing data

For 18S data, sequences were trimmed and binned into OTUs similar to that of 16S data. Each representative sequence was assigned to taxonomy against a subset of Silva 104 database (http://www.arb-silva.de/download/archive/qiime/). The OTUs defined at 97% similarity were used to perform rarefaction analysis and to calculate the richness and diversity indices. Representative sequences of the most 20 abundant OTUs were then searched for the best BLAST hit on NCBI. Representative sequence that was assigned to previously reported genus with taxol-production capacity species (S1 Table) was blasted in GenBank for further species level taxonomy identification. The pyrosequencing reads have been deposited at GenBank with accession number SRP040943.

### Metagenomic analysis

Sequences from host plant were filtered using SOAPaligner (Version 2.21, http://soap.genomics.org.cn/soapaligner.html) with a match requirement of 95% sequence identity. Reads were assembled by IDBA_UD (http://i.cs.hku.hk/~alse/hkubrg/projects/idba_ud/index.html) with k value between 31 and 61 with a step of 10. Gene taxonomic assignments were made on the basis of BLASTP search (e-value < 10^−5^) of the NCBI-NR database. Gene functional annotations were made by BLASTP [43] search (E-value < 10^−5^) with eggNOG and KEGG (v48.2) databases.

### Availability of supporting data

The pyrosequencing reads have been deposited were deposited in GenBank (NCBI) under the accession numbers SRP040943.

## Supporting Information

S1 Table List of reported microbes with taxol production capability. (DOCX)

S2 Table List of the 13 reported taxol biosynthetic enzymes in Taxus sp. (DOC)

S3 Table List of bacterial genus and reads number in meta-genome sequencing. (XLS)

S4 Table List of fungal genus and reads number in meta-genome sequencing. (XLS)

S5 Table List of gene annotation with IDBA_UD assembly. (XLSX)

## References

[1] Suffness M, Wall ME. Discovery and development of taxol In: Suffness M (ed) Taxol science and applications. CRC Press, Boca Raton 1995pp 3–25

[2] Arbuck SG, Blaylock BA. Taxol: clinical results and current issues in development In: Suffness M (ed), Taxol: Science and Applications. CRC Press, Boca Raton, Florida 1995 pp. 379–415.

[3] Wall ME, Wani MC. Paclitaxel: from discovery to clinic. In: Georg GI, Chen TT, Ojima I, Vyas DM (eds), Taxane Anticancer Agents: Basic Science and Current Status ACS Symposium Series, 583. 1995. pp. 18–30.

[4] Strobel GA, Hess WM, Ford E, Sidhu RS, Yang X. Taxol from fungal endophytes and the issue of biodiversity. J Ind Microbiol 1996;17: 417–423.

[5] Stierle A, Strobel G, Stierle D. Taxol and taxane production by *Taxomyces andreanae*, an endophytic fungus of Pacific yew. Science. 1993; 260: 214–216. 8097061 10.1126/science.8097061

[6] Rivera-Orduña FN, Suarez-Sanchez RA, Flores-Bustamante ZR, Gracida-Rodriguez JN, Flores-Cotera LB. Diversity of endophytic fungi of *Taxus globosa* (Mexican yew). Fungal Divers. 2011; 47: 65–74.

[7] Kumar DDS, Hyde KD. Biodiversity and tissue-recurrence of endophytic fungi in *Tripterygium wilfordii* . Fungal Divers. 2004; 7:69–90.

[8] Huang WY, Cai YZ, Surveswaran S, Hyde KD, Corke H, Sun M. Molecular phylogenetic identification of endophytic fungi isolated from three Artemisia species. Fungal Divers. 2009; 36:69–88.

[9] Lin X, Huang YJ, Zheng ZH, Su WJ, Qian XM, Shen YM. Endophytes from the pharmaceutical plant, *Annona squamosa*: isolation, bioactivity, identification and diversity of its polyketide synthase gene. Fungal Divers. 2010; 41:41–51.

[10] Kang JC, Jin R, Wen T, He J, Lei BX. Recent research advances on endophytic fungi producing taxol. Mycosystema 2011; 30: 168–179.

[11] Lou J, Niu XL, Yan F, Pan J, Zhu XD. Recent progresses in the studies of taxol and taxane-producing fungi. Mycosystema 2011; 30: 158–167.

[12] Flores-Bustamante ZR, Rivera-Orduna FN, Martinez-Cardenas A, Flores-Cotera LB. Microbial paclitaxel: advances and perspectives. J Antibiot. 2010; 63:460–467. 10.1038/ja.2010.83 20628412

[13] Ajikumar PK, Xiao WH, Tyo KE, Wang Y, Simeon F, Leonard E, Mucha O, Phon TH, Pfeifer B, Stephanopoulos G. Isoprenoid pathway optimization for Taxol precursor overproduction in *Escherichia coli* . Science 2010; 330:70–74. 10.1126/science.1191652 20929806 PMC3034138

[14] Zhao K, Ping WX, Zhang LN, Liu J, Lin Y, Jin T, et al Screening and breeding of high taxol producing fungi by genome shuffling. Sci China Ser C: Life Sci. 2008; 51:222–231. 18246310 10.1007/s11427-008-0037-5

[15] Wang Y, Guo B, Miao Z, Tang K. Transformation of taxol-producing endophytic fungi by restriction enzyme-mediated integration (REMI). FEMS Microbiol Lett. 2007; 273:253–259. 17608701 10.1111/j.1574-6968.2007.00801.x

[16] Li JY, Sidhu RS, Bollon A, Strobel GA. Stimulation of taxol production in liquid cultures of *Pestalotiopsis microspora* . Mycol Res. 1998; 102:461–464.

[17] Xiong ZQ, Yang YY, Zhao N, Wang Y. Diversity of endophytic fungi and screening of fungal paclitaxel producer from Anglojap yew, *Taxus* x *media* . BMC Microbiol. 2013; 13:71 10.1186/1471-2180-13-71 23537181 PMC3618195

[18] Zhang P, Zhou PP, Jiang C, Yu H, Yu LJ. Screening of taxol-producing fungi based on PCR amplification from *Taxus* . Biotechnol Lett. 2008; 30:2119–2123. 10.1007/s10529-008-9801-7 18709488

[19] Staniek A, Woerdenbag HJ, Kayser O. *Taxomyces andreanae*: a presumed paclitaxel producer demystified? Plant Med. 2009; 75:1–6. 10.1055/s-0029-1186181 19809969

[20] Miao Z, Wang Y, Yu X, Guo B, Tang K. A new endophytic taxane producing fungus from *Taxus chinensis* . Appl Biochem Micobiol. 2009; 45:81–86. 19235515

[21] Kumaran RS, Kim HJ, Hur BK. Taxol promising fungal endophyte, Pestalotiopsis species isolated from *Taxus cuspidata* . J Biosci Bioeng. 2010; 110:541–546. 10.1016/j.jbiosc.2010.06.007 20634132

[22] Zhou X, Wang Z, Jiang K, Wei Y, Lin J, Sun X, et al Screening of taxol-producing endophytic fungi from *Taxus chinensis* var. *mairei* . Appl Biochem Microbiol. 2007; 43:439–443. 17929579

[23] Yang YF, Zhao HN, Barrero RA, Zhang BH, Sun GL, Wilson IW, et al Genome sequencing and analysis of the paclitaxel-producing endophytic fungus *Penicillium aurantiogriseum* NRRL 62431. BMC Genomics 2014; 15:69 10.1186/1471-2164-15-69 24460898 PMC3925984

[24] Lundberg DS, Lebeis SL, Paredes SH, Yourstone S, Gehring J, Malfatti S, et al Defining the core *Arabidopsis thaliana* root microbiome. Nature 2012; 488:86–90. 10.1038/nature11237 22859206 PMC4074413

[25] Bulgarelli D, Rott M, Schlaeppi K, Ver Loren van Themaat E, Ahmadinejad N, Assenza F, et al Revealing structure and assembly cues for Arabidopsis root-inhabiting bacterial microbiota. Nature 2012; 488(7409):91–95. 10.1038/nature11336 22859207

[26] Caruso M, Colombo AL, Fedeli L, Pavesi A, Quaroni S, Saracchi M, et al Isolation of endophytic fungi and actinomycetes. Ann Microbiol. 2000; 50(1): 3–13.

[27] Wang YT, Lo HS, Wang PH. Endophytic fungi from *Taxus mairei* in Taiwan: first report of *Colletotrichum gloeosporioides* as an endophyte of *Taxus mairei* . Bot Stud. 2008; 49: 39–43.

[28] Liu K, Ding X, Deng B, Chen W. Isolation and characterization of endophytic taxol-producing fungi from *Taxus chinensis* . J Ind Microbiol Biotechnol. 2009; 36:1171–1177. 10.1007/s10295-009-0598-8 19484278

[29] Page M, Landry N. Bacterial mass production of taxanes with *Erwinia*. US Patent 5561055, 1996.

[30] Hanson RL, Howell JM, Brzozowski DB, Sullivan SA, Patel RN, Szarka LJ. Enzymatic hydrolysis of 7-xylosyltaxanes by xylosidase from *Moraxella* sp. Biotechnol Appl Biochem. 1997; 26:153–158.

[31] Hao DC, Ge GB, Yang L. Bacterial diversity of *Taxus* rhizosphere: culture-independent and culture-dependent approaches. FEMS Microbiol Lett. 2008; 284:204–212. 10.1111/j.1574-6968.2008.01201.x 18576948

[32] Axelrood PE, Chow ML, Radomski CC, McDermott JM, Davies J. Molecular characterization of bacterial diversity from British Columbia forest soils subjected to disturbance. Can J Microbiol. 2002; 8(7):655–674. 10.1139/w02-059 12224564

[33] Filion M, Hamelin RC, Bernier L, St-Arnaud M. Molecular profiling of rhizosphere microbial communities associated with healthy and diseased black spruce (Picea mariana) seedlings grown in a nursery. Appl Environ Microbiol. 2004; 70(6):3541–3551. 15184155 10.1128/AEM.70.6.3541-3551.2004 PMC427751

[34] Croteau R, Ketchum RE, Long RM, Kaspera R, Wildung MR. Taxol biosynthesis and molecular genetics. Phytochem Rev. 2006; 5:75–97. 20622989 10.1007/s11101-005-3748-2 PMC2901146

[35] Zhang P, Zhou P-P, Yu L-J. An endophytic taxol-producing fungus from *Taxus media*, *Cladosporium cladosporioides* MD2. Curr Microbiol. 2009; 59:227–232. 10.1007/s00284-008-9270-1 19484305

[36] Zhang P, Zhou P-P, Yu L-J. An endophytic taxol-producing fungus from *Taxus* x *media*, *Aspergillus candidus* MD3. FEMS Microbiol Lett. 2009; 293:155–159. 10.1111/j.1574-6968.2009.01481.x 19239498

[37] Mirjalili MH, Farzaneh M, Bonfill M, Rezadoost H, Ghassempour A. Isolation and characterization of Stemphylium sedicola SBU-16 as a new endophytic taxol-producing fungus from Taxus baccata grown in Iran. FEMS Microbiol Lett. 2012; 328:122–129. 10.1111/j.1574-6968.2011.02488.x 22211912

[38] Li YC, Tao WY. Interactions of taxol-producing endophytic fungus with its host (*Taxus* spp.) during taxol accumulation. Cell Biol Int. 2009; 33:106–112. 10.1016/j.cellbi.2008.10.007 18996212

[39] Soliman SS, Trobacher CP, Tsao R, Greenwood JS, Raizada MN. A fungal endophyte induces transcription of genes encoding a redundant fungicide pathway in its host plant. BMC Plant Biol. 2013; 13:93 10.1186/1471-2229-13-93 23802696 PMC3700885

[40] Soliman SSM, Raizada MN. Interactions between co-habitating fungi elicit synthesis of taxol from an endophytic fungus in host *Taxus* plants. Front Microbio. 2013; 4:3. 10.3389/fmicb.2013.00003 PMC3550802 23346084

[41] Malik S, Cusidó RM, Mirjalili MH, Moyano E, Palazón J, Bonfill M. Production of the anticancer drug taxol in Taxus baccata suspension cultures: A review.Process Bioche 2011;46:23–34.

[42] Sul WJ, Cole JR, Jesus EDC, Wang Q, Farris RJ, Fish A, et al Bacterial community comparisons by taxonomy-supervised analysis independent of sequence alignment and clustering. Proc Natl Acad Sci USA. 2011; 108: 14637–14642. 10.1073/pnas.1111435108 21873204 PMC3167511

[43] Altschul SF, Madden TL, Schäffer AA, Zhang J, Zhang Z, Miller W, et al Gapped BLAST and PSI-BLAST: a new generation of protein database search programs. Nucleic Acids Res. 1997; 25(17):3389–3402. 9254694 10.1093/nar/25.17.3389 PMC146917

