# Supplementary material for: Baseline Survey of Root-Associated Microbes of Taxus chinensis (Pilger) Rehd
Source: PLoS One. 2015 Mar 30;10(3):e0123026. doi: 10.1371/journal.pone.0123026 (PMC4378922; doi:10.1371/journal.pone.0123026)
Supplement: S1 Table — (DOCX) [file pone.0123026.s001.docx]

Table S1 List of reported microbes with taxol production capability.

| No. | taxa | reference |
| --- | --- | --- |
| 1 | Botryosphaeria sp. | Giovanny et al. 2011, Fungal Biology |
| 2 | Pleosporales | Giovanny et al. 2011, Fungal Biology |
| 3 | Penicillium sp. | Giovanny et al. 2011, Fungal Biology |
| 4 | Gyromitra sp. | Giovanny et al. 2011, Fungal Biology |
| 5 | Acremonium sp. | Giovanny et al. 2011, Fungal Biology |
| 6 | Fusarium sp. | Giovanny et al. 2011, Fungal Biology |
| 7 | Fusarium sp. | Giovanny et al. 2011, Fungal Biology |
| 8 | Fusarium sp. | Giovanny et al. 2011, Fungal Biology |
| 9 | Nigrospora sp. | Giovanny et al. 2011, Fungal Biology |
| 10 | Nigrospora sp. | Giovanny et al. 2011, Fungal Biology |
| 11 | Xylaria sp. | Giovanny et al. 2011, Fungal Biology |
| 12 | Xylaria sp. | Giovanny et al. 2011, Fungal Biology |
| 13 | Alternaria sp. | Caruso et al. 2000, Annals of Microbiology |
| 14 | Aspergillus sp. | Caruso et al. 2000, Annals of Microbiology |
| 15 | Beauveria sp. | Caruso et al. 2000, Annals of Microbiology |
| 16 | Epicoccum sp. | Caruso et al. 2000, Annals of Microbiology |
| 17 | Fusarium sp. | Caruso et al. 2000, Annals of Microbiology |
| 18 | Gelasinospora sp. | Caruso et al. 2000, Annals of Microbiology |
| 19 | Geotrichum sp. | Caruso et al. 2000, Annals of Microbiology |
| 20 | Phoma sp. | Caruso et al. 2000, Annals of Microbiology |
| 21 | Phomopsis sp. | Caruso et al. 2000, Annals of Microbiology |
| 22 | Mycelia sterilia | Caruso et al. 2000, Annals of Microbiology |
| 23 | Colletotrichum gloeosporioides | Xiong et al. 2013, BMC Microbiology |
| 24 | Guignardia mangiferae | Xiong et al. 2013, BMC Microbiology |
| 25 | Fusarium proliferatum | Xiong et al. 2013, BMC Microbiology |
| 26 | Fusarium solani | Chakravarthi et al. 2008, Journal of Biosciences |
| 27 | Gliocladium sp. | Sreekanth et al. 2009, Journal of Microbiology and Biotechnology |
| 28 | Aspergillus flavus | Banu and Muthumary 2010, Health |
| 29 | Bartalinia robillardoides | Gangadevi and Muthumary 2008, World Journal of Microbiology and Biotechnology |
| 30 | Pestalotiopsis microspora | Strobel et al. 1996, Microbiology |
| 31 | Phyllosticta tabernaemontanae | Kumaran et al. 2009, The Journal of Microbiology |
| 32 | Phyllosticta citricarpa | Kumaran et al. 2008, Journal of Bioscience and Bioengineering |
| 33 | Colletotrichum capsici | Kumaran et al. 2011, Engineering in Life Sciences |
| 34 | Pestalotiopsis terminaliae | Gangadevi 2009, Biotechnology and Applied Biochemistry |
| 35 | Pestalotiopsis malicola | Bi et al. 2011, African Journal of Biotechnology |
| 36 | Metarhizium anisopliae | Liu et al. 2009, Journal of Industrial Microbiology & Biotechnology |
| 37 | Monochaetia sp. | Strobel et al. 1996b, Journal of Industrial Microbiology |
| 38 | Mycelia sterillia | Guo et al. 2007, Acta Botanica Boreali |
| 39 | Papulaspora sp. | Hu et al. 2006, Journal of Southwest China Normal University |
| 40 | Achaetomium sp. | Guo et al. 2007, Acta Botanica Boreali |
| 41 | Botrytis sp. | Hu et al. 2006, Journal of Southwest China Normal University |
| 42 | Cheatomella raphigera | Gangadevi et al. 2009, Applied Biochemistry and Biotechnology |
| 43 | Ectostroma sp. | Hu et al. 2006, Journal of Southwest China Normal University |
| 44 | Periconia sp. | Li et al. 1998, Journal of Industrial Microbiology & Biotechnology |
| 45 | Pestalotia bicilia | Strobel et al. 1996b, Journal of Industrial Microbiology |
| 46 | Pithomyces sp. | Strobel et al. 1996b, Journal of Industrial Microbiology |
| 47 | Taxomyces andreanae | Stierle et al. 1993, Science |
| 48 | Tubercularia sp. | Wang et al. 1999, Journal of Xiamen University |
| 49 | Bionectria | Yu and Hu 2007, Journal of Southwest University |
| 50 | Botryodiplodia theobromae | Venkatachalam et al. 2008, Journal of Biotechnology |
| 51 | Chaetomium | Chen et al. 2003, Biotechnology |
| 52 | Cladosporium cladosporioides | Zhang et al. 2008b, Biotechnological Letter |
| 53 | Conia sp. | Li et al. 1998, Journal of Industrial Microbiology & Biotechnology |
| 54 | Mucor sp. | Chen et al. 2003, Biotechnology |
| 55 | Phoma sp. | Chen et al. 2003, Biotechnology |
| 56 | Phomopsis sp. | Kumaran & Hur 2009, Applied Biochemistry Biotechnology |
| 57 | Rhizoctonia sp. | Chen et al. 2003, Biotechnology |
| 58 | Trichoderma sp. | Chen et al. 2003, Biotechnology |
| 59 | Alemaria sp. | Strobel et al. 1996b, Journal of Industrial Microbiology |
| 60 | Erwinia (Sphingomonas) sp. | Landry and Page patent US5561055 A |
