# Supplementary material for: Baseline Survey of Root-Associated Microbes of Taxus chinensis (Pilger) Rehd
Source: PLoS One. 2015 Mar 30;10(3):e0123026. doi: 10.1371/journal.pone.0123026 (PMC4378922; doi:10.1371/journal.pone.0123026)
Supplement: S2 Table — (DOC) [file pone.0123026.s002.doc]

**Table S2.** List of the 13 reported taxol biosynthetic enzymes in *Taxus* sp.

| **Enzyme** | **Abbreviations** | **Genbank Accession No.** | **CDS**  **(bp)** | **Reference** |
| --- | --- | --- | --- | --- |
| Baccatin III: 3-amino-3-phenylpropanoyltransferase | BAPT | AY082804 | 1,335 | Walker et al., 2002a |
| 10-deacetylbaccatin III-10-O-acetyltransferase | DBAT | AF193765 | 1,320 | Walker et al., 2000c |
| 3′-N-debenzoyl-2′-deoxytaxol N-benzoyltransferase | DBTNBT | AF466397 | 1,323 | Walker et al., 2000b |
| Geranylgeranyl diphosphate synthase | GGPPS | AF081514 | 1,182 | Hefner et al., 1998 |
| Phenylalanine aminomutase | PAM | AY582743 | 2,094 | Walker et al., 2004 |
| Taxane 10-beta hydroxylase | T10H | AF318211 | 1,494 | Schoendirf et al., 2001 |
| Taxane 13-alpha hydroxylase | T13H | AY056019 | 1,458 | Jennewein et al., 2001 |
| Taxane 2-alpha hydroxylase | T2H | AY518383 | 1,488 | Chau et al., 2004a |
| Taxane 5-alpha hydroxylase | T5H | AY307951 | 1,503 | Chau et al., 2004b |
| Taxane 7-beta hydroxylase | T7H | AY289209 | 1509 | Jennewein et al., 2004 |
| Taxadien-5α-ol-O-acetyl transferase | TAT | AF190130 | 1,317 | Walker et al., 2000a |
| Taxane 2α-O-benzoyltransferase | TBT | AF297618 | 1,320 | Walker et al., 2000b |
| Taxadiene synthase | TS | AY364469 | 2,586 | Wildung et al., 1996 |
| Taxadiene synthase | TS | U48796 | 2586 | Wildung et al., 1996 |
